# Cancer as an infective disease: the role of EVs in tumorigenesis

**DOI:** 10.1002/1878-0261.13316

**Published:** 2022-10-12

**Authors:** Lucia Robado de Lope, Estela Sánchez‐Herrero, Roberto Serna‐Blasco, Mariano Provencio, Atocha Romero

**Affiliations:** ^1^ Liquid Biopsy Laboratory Biomedical Sciences Research Institute Puerta de Hierro‐Majadahonda Spain; ^2^ Atrys Health Barcelona Spain; ^3^ Medical Oncology Department Hospital Universitario Puerta de Hierro‐Majadahonda Spain

**Keywords:** cancer, extracellular vesicles, metastasis, oncogenic transformation

## Abstract

Cancer is conventionally considered an evolutionary disease where tumor cells adapt to the environment and evolve eventually leading to the formation of metastasis through the seeding and growth of metastasis‐initiating cells in distant organs. Tumor cell and tumor‐stroma communication via soluble factors and extracellular vesicles (EVs) are essential for the success of the metastatic process. As the field of EVs advances, growing data support the role of tumor‐derived EVs not only in modifying the microenvironment to facilitate tumor progression but also in inducing changes in cells outside the primary tumor that may lead to a malignant transformation. Thus, an alternative hypothesis has emerged suggesting the conceptualization of cancer as an ‘infective’ disease. Still, tackling EVs as a possible cancer treatment has not been widely explored. A major understanding is needed to unveil possible additional contributions of EVs in progression and metastasis, which may be essential for the development of novel approaches to treat cancer patients. Here, we review the contribution of EVs to cancer progression and the possible implication of these factors in the oncogenic transformation of indolent cells.

## Graphical abstract

Intercellular communication via extracellular vesicles (EVs) has acquired increasing importance due to EV roles in multiple steps of cancer progression and metastasis. Here, we review the contribution of EVs to cancer progression and the possible implication of these factors in the oncogenic transformation of indolent cells, which may lead to the conceptualization of cancer as an ‘infective’ disease.

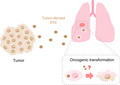

**Abbreviations**
CAFs: cancer‐associated fibroblasts
circRNAs: circular RNAs
CRC: colorectal cancer
ECM: extracellular matrix
EGFRvIII: epidermal growth factor receptor variant III
EMT: epithelial‐mesenchymal transition
EVs: extracellular vesicles
HMECs: human primary mammary epithelial cells
ITH: intratumor heterogeneity
L‐EVs: large EVs
lncRNAs: long noncoding RNAs
MICs: metastasis‐initiating cells
miRNAs: microRNAs
NK: natural killer
NSCLC: non‐small cell lung carcinoma
PMN: premetastatic niche
sEVs: small EVs

## 1 Introduction

Cancer is a leading cause of morbidity and mortality worldwide with nearly 19.3 million new cases and 10 million cancer‐associated deaths in 2020 [1]. Critically, cancer incidence is estimated to increase by 47% in 2040 [1], which manifests the magnitude of the social problem that cancer represents. To this extent, it is crucial to understand tumor evolution and progression, together with the complex interactions between tumor cells and the surrounding microenvironment.

In this sense, extensive research has been performed in order to understand how this process develops.

## 2 Cancer origin and evolution

Cancers are proposed to originate from the malignant transformation of normal tissue cells [2]. Once the cancer cell of origin is transformed, it starts to proliferate abnormally, finally leading to the development of a tumor [3]. The mechanisms by which this process occurs have been extensively discussed and encompassed different theories including the occurrence of multiple mutations, chromosomal imbalance, mitochondrial dysfunction, or microenvironmental changes, but any of them can be exclusively proposed as the cause of cancer initiation and progression, the interplay of all those factors being the most probable hypothesis [4].

Indeed, several milestones completely changed the history of cancer research in the last decades and enabled a better understanding of the origin of cancer. The observation of the link between viruses and human cancer [5], the implication of chromosomal abnormalities in cancer development [6], the identification of oncogenes [7], and the importance of the host microenvironment [8] are just some examples of the key discoveries that envisioned the complexity of this disease.

Focusing on the genetic bases of cancer, in the 1970s, Nowell, Battula and collaborators proposed that loss of DNA repair genes and acquired genomic alterations led to cellular transformation [9, 10]. Since then, several studies have focused on the understanding of genomic instability, identifying mutational signatures and their contribution to the development of different cancer types [11, 12, 13, 14, 15, 16, 17, 18].

Accumulating evidence reveals that genetic diversity within tumors is a key factor associated with increased risk of recurrence or death among cancer patients [19] along with therapy resistance [20]. One of the theories that can explain the existence of this heterogeneity is the clonal evolution model of tumor cell populations described by Nowell in 1976, who proposed that tumors arise from a single cell of origin that proliferate and acquire genetic variability allowing the sequential selection of more favorable clones [9]. Since then, countless studies reported that intratumor heterogeneity (ITH), mainly driven by genetic instability (chromosomal instability, somatic mutations) and epigenetic changes, can act as a substrate for tumor evolution [21] and enable the adaptation of tumor cells to different selective pressures such as immune surveillance, colonization of novel niches and therapy evasion. Indeed, a huge effort to understand tumor evolution is being developed with a recent collaborative project known as TRACERx (Tracking Cancer Evolution through Therapy), which attempts to evaluate the relationship between ITH and patient outcome, monitoring the process as tumor progresses [22, 23] (refer to [22] for further information).

Thus, cancer development and progression have mainly been considered as evolutionary processes by which heterogeneous populations of tumor cells are selected in every step of carcinogenesis, from tumor formation to metastasis [24]. Still, the selection is not always operational, as it depends on the environmental context. Hence, a process of neutral evolution occurs between selective events, in which tumor cell populations grow and experience mutation and drift (stochastic processes that can change subclone frequency), without affecting their fitness [25]. Thus, both selection and neutral evolution may be operating simultaneously within the same tumor [21]. Recently, other models of evolution such as the theory of multi‐task evolution have also been proposed. This theory considers tumors as a ‘whole ecosystem’ that can specialize in one or more tasks, and enables the identification of different trade‐offs, providing a specific structure for tumor diversity and helping to interpret ITH and spatial architecture of tumors. Thus, driver mutations will tune the gene expression profile of the tumors toward specialization in a particular task, enabling the selection of trade‐offs and the maximization of total tissue performance [26].

Besides genetic alterations in tumor cells, the presence of an aberrant tumor microenvironment is considered a crucial element for tumor progression, influencing malignant cell phenotype and contributing to the selection of cancer cells under different selective pressures [27, 28]. Indeed, the continuous interplay between cancer cells and cells within the tumor microenvironment leads to tumor‐stroma co‐evolution, triggering a dynamic balance that supports tumor growth, local invasion, and metastatic dissemination [28, 29]. The composition of the tumor microenvironment is characteristic of each tumor type, containing distinctive elements including immune cells, endothelial cells, cancer‐associated fibroblasts (CAFs), and noncellular components such as the extracellular matrix (ECM) [27, 29]. Furthermore, the secretion of soluble factors (cytokines, chemokines, growth factors) also plays a determinant role in disease progression, enabling tumor‐stroma communication at the local niche and in distal sites [30]. Once tumors start to grow, the absence of oxygen and nutrients activate an angiogenic switch that helps to provide tumor cells with essential requirements to continue growing and facilitates the subsequent spreading of cancer cells [27, 29, 31].

Indeed, tumor cell dissemination can start early during disease progression [32, 33, 34, 35, 36], although the majority of cells that leave the primary tumor do not succeed in the formation of distant lesions, making metastasis a highly inefficient process [37]. During the ‘metastatic cascade’ cancer cells need to overcome different steps including invasion through the stroma, intravasation into the bloodstream and survival in circulation, extravasation into the parenchyma of target organs, acquisition of immune resistance, adaptation to supportive niches, achievement of long‐term survival during a dormant state, and finally, metastatic outgrowth [37, 38]. Interaction with the host microenvironment is essential to successfully complete all the steps of this process. The small proportion of tumor cells that are able to survive the stresses of the metastatic process is called metastasis‐initiating cells (MICs), which share a characteristic stem‐like phenotype defined by the ability to regenerate tumors in distant locations and the capacity to evolve during the metastatic process with a high grade of cellular plasticity [38, 39]. The mechanisms underlying metastasis‐initiating capacity are not well understood. Still, a recent publication by Ganesh and collaborators defined L1 cell adhesion molecule (L1CAM) expression as a fundamental requirement for metastatic tumor regrowth [40].

**Fig. 1:**
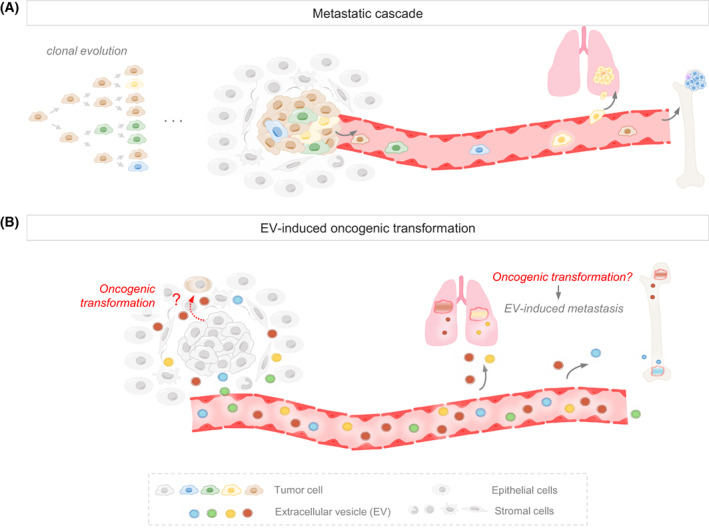
Metastatic process. (A) Metastatic cascade. During metastasis, tumor cells with different properties leave the primary tumor and disseminate throughout the body seeding multiple organs. Once in the target organs, metastatic cells need to exit dormancy to grow and finally form a macrometastasis. (B) Alternative metastatic process. EVs secreted from the primary tumor are taken up by cells from local and distant microenvironments, which may induce oncogenic transformation leading to the formation of EV‐induced metastasis.

In brief, cancer is perceived as an evolutionary process, and it is generally believed that MICs are responsible for the colonization of distant organs and the formation of metastasis (Fig. 1A). However, as mentioned above, tumor‐stroma cells interactions in both primary and metastatic lesions are essential to facilitate tumor progression, and may be mainly accomplished by secreted molecules. Although soluble factors were first described as mediators of this communication, extracellular vesicles (EVs) are gaining increasing importance in this regard. The conceptualization of cancer as an ‘infective’ disease is less accepted (Fig. 1B). However, there is growing evidence indicating that, in cancer patients, plasma is flooded with millions of vesicles, molecules such as ctDNA, and soluble factors that may have an underestimated role in the pathogenesis of cancer.

## 3 EVs, key mediators of cell–cell communication

Extracellular vesicles are lipid bilayer‐delimited particles released from most cells that comprise a heterogeneous population with differences in biogenesis, size, and content [41, 42]. A classification based on size groups them into either large EVs (L‐EVs) or small EVs (sEVs) [41, 42]. L‐EVs include vesicles classically known as oncosomes, apoptotic bodies, and microvesicles; whereas exosomes are classified as sEVs.

Although the term ‘extracellular vesicle’ has now been agreed on as the consensus generic term by the international community, EV nomenclature has created confusion in the field, suggesting the need to report a minimal set of characterizing features to describe them [42]. Hence, we will use the established term in each publication when describing previous work, and the consensus term for general and original ideas.

Extracellular vesicles are produced in normal cell physiology and in many pathological conditions, including cancer. Notably, they carry a representative molecular cargo of the cell of origin that includes proteins (enzymes, transcription factors, ECM proteins), nucleic acids (DNA, mRNA, noncoding RNAs), and lipids, which can modulate the phenotype of recipient cells by the transmission of factors or the induction of intracellular signaling through direct binding to cell receptors [43, 44, 45, 46]. Hence, EVs derived from normal or pathology‐associated cells exhibit a unique molecular signature that differentially affects cellular behavior in recipient cells.

In physiological conditions, EVs have been proposed as regulators of cellular and tissue balance and homeostasis, acting together with endocrine and paracrine signals [47]. They are involved in the control of main signaling pathways and processes such as coagulation and hemostasis, angiogenesis, innate and acquired immunity, embryogenesis or tissue repair, and the transfer of information between different cell types [48]. However, although some insights regarding the role of EVs in physiological conditions have been described, efforts have mainly focused on elucidating the role of EVs in different pathological processes such as cancer or neurological disorders. Indeed, EVs are involved in multiple steps of tumor progression [49] including the modification of the tumor microenvironment [50, 51, 52, 53], premetastatic niche (PMN) formation, [54] or the acquisition of malignant properties in tumor cells [55].

Here, we will discuss the role of EVs in tumor development and progression, focusing on the potential role of these vesicles in the transformation of tumor and microenvironmental cells.

## 4 EVs and cancer, an intimate relationship

**Fig. 2:**
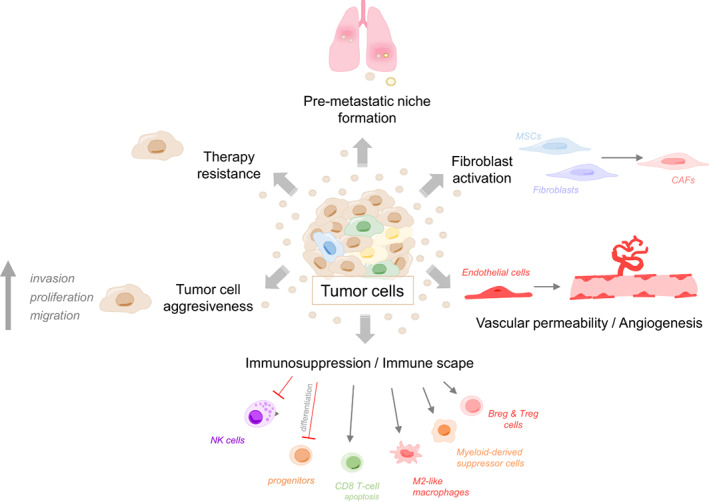
Role of EVs in cancer development and progression. EVs are essential for tumor progression and metastasis facilitating immunosuppression and PMN formation together with the induction of changes in stromal cells from the microenvironment. They promote fibroblast activation toward CAFs, vascular permeability, and angiogenesis. Furthermore, EVs are also involved in the modification of tumor cells phenotype, inducing therapy resistance and tumor cell aggressiveness. cancer‐associated fibroblasts, CAFs; Extracellular vesicles, EVs; mesenchymal stem cells, MSCs.

During cancer development and progression, tumor cells communicate with local and distal microenvironments promoting the metastatic process. Although soluble factors were traditionally identified as key players of this crosstalk, EVs are emerging as crucial mediators in the communication between tumor and stromal cells [49]. Indeed, like soluble factors, EVs can act both locally and distally during tumor progression, transferring their cargo to recipient cells and favoring tumor growth, PMN formation, and, eventually, metastasis [56, 57]. Thus, EV‐based communication has arisen as a key mechanism involved in tumor progression and metastatic spread [49] (Fig. 2).

### 4.1 Role of EVs at primary sites

Tumor cells from primary sites release EVs that are transferred to other tumor cells and cells from the tumor microenvironment, both being crucial for tumor success. EVs can carry oncogenic proteins and RNA that induce, among others, proliferation, inhibition of apoptosis, and invasive potential in cancer recipient cells [52, 55, 58]. For instance, the intercellular exchange of the epidermal growth factor receptor variant III (EGFRvIII) between glioma cells by tumor microvesicles transfers oncogenic activity to cancer cells lacking EGFRvIII, stimulating tumor cell proliferation via the activation of MAPK and AKT signaling pathways [55]. Likewise, it has been reported that exosomes from *KRAS* mutant colorectal cancer (CRC) cells transfer mutant *KRAS* to nonmutated cells enhancing three‐dimensional growth [59]. In lung cancer models, activation of the c‐Met cascade in poorly metastatic cells by the uptake of hepatocyte growth factor‐sEVs from cells with more aggressive potential promotes tumor cell migration and epithelial‐mesenchymal transition (EMT) [60]. Importantly, *in vivo* models using the Cre‐LoxP system enable the discrimination of cells that take or do not take up EVs [61, 62]. This technique allowed the observation of an enhanced migratory behavior and metastatic capacity of low malignant breast cancer cells in living mice after the uptake of EVs derived from more aggressive tumor cells [62]. Using the same approach, Steenbeek et al. [63] described that the *in vivo* exchange of EVs between syngeneic melanoma cells (B16) with different metastatic potential increased the migratory behavior of the less metastatic ones, through the transfer of different proteins and RNAs from interconnected signaling networks involved in migration, among other processes. Furthermore, in pancreatic ductal adenocarcinoma tumors, an organized and plastic cooperation network known as EVnet has been identified, by which subpopulations of cancer cells communicate through EVs (preferentially from cancer stem cells to non‐stem cancer cells), allowing tumors to adapt and thrive [64]. Together, these studies demonstrate that tumor cells can phenocopy the behavior of more aggressive counterparts through the exchange of EVs, which induce molecular alterations and morphological transformation.

Extracellular vesicles also play a role in shaping the local microenvironment. Activation of fibroblasts or mesenchymal stem cells into CAFs, induction of angiogenesis, and evasion of local immunity are just some insights into the whole picture of microenvironmental changes affected by tumor‐derived EVs. For instance, exosomal TGF‐β, miR‐10b, miR‐1247‐3p, and miR‐125b secreted by bladder, colorectal, liver, and breast cancer tumor cells, respectively, mediate the activation of CAFs [65, 66, 67, 68], which, in turn, promote tumor cells aggressiveness through the induction of proliferation, EMT, stemness, chemoresistance and/or tumorigenicity, among others [66, 67]. These effects are observed in multiple tumor types [69, 70, 71, 72], considering CAFs as important stromal cells contributing to tumor progression [73, 74, 75]. Nevertheless, some publications described an antitumorigenic role of some populations of CAFs (reviewed in Refs [76, 77]). Still, as far as we know, there is no evidence to support the antitumor effect of CAF‐derived EVs. Endothelial cells can also uptake tumor‐derived EVs resulting in the activation of angiogenesis. The horizontal transfer of VEGF or specific molecules regulating the VEGF pathway, together with factors not involved in the stimulation of VEGF, contribute to this phenotype in diverse tumor types [78]. For example, lncRNA CCAT2 delivered in EVs from U87‐MG glioma cells upregulated VEGF‐A and TGF‐β in HUVECs and promoted HUVEC migration, proliferation, tubular‐like structure formation *in vitro*, and arteriole formation *in vivo* in addition to the inhibition of HUVEC apoptosis induced by hypoxia [79]. Similarly, CCA‐associated circRNA 1 (circ‐CCAC1) increased cell leakiness and induced angiogenesis in endothelial cells [80] and miR‐130a has been described as an important promoter of tumor angiogenesis in gastric cancer [81]. In line with these data, tumor‐derived EVs are involved in the generation of immunosuppressive microenvironments that protect the tumor from the immune system, impairing both the innate and the adaptive responses [82]. In particular, EVs can inhibit the differentiation of myeloid and lymphoid progenitors and dendritic cell maturation, boost the polarization of macrophages toward an M2‐like tumor‐associated phenotype, promote the expansion and reprogramming of myeloid‐derived suppressor cells to elicit immunosuppressive functions, inhibit natural killers (NK) cells, induce CD8^+^ T‐cell apoptosis, and stimulate the expansion of Treg and Breg cells [82]. For instance, EVs derived from multiple tumor types including melanoma, glioblastoma, gastric cancer, and osteosarcoma can induce the expression of PD‐L1 in different immune cells such as monocytes or neutrophils, which help to suppress T‐cell immunity [83, 84]. Importantly, PDL‐1 can be also transported in tumor‐derived EVs and bind directly to PD‐1 receptors on T‐cells, suppressing their activation and inhibiting their killing activity [85, 86, 87, 88, 89, 90] (extended review [91]).

Collectively, all these studies strongly support the role of EVs derived from tumor cells as active participants priming surrounding cells within the tumor microenvironment, finally leading to tumor growth and progression.

### 4.2 EVs, premetastatic niche formation, metastatic outgrowth, and cancer therapeutics

In recent years, EVs have been described as crucial mediators of metastasis influencing all steps of tumor cell dissemination including the establishment of the PMN, local invasion, colonization of sentinel lymph nodes, and finally the development of distal metastasis [56, 92].

Pioneering studies from Kaplan et al. [93] demonstrated that tumor cells induce the formation of PMNs at distant organs that facilitate metastatic cell survival and outgrowth prior to their arrival at these sites. Multiple soluble factors and EVs have been identified as mediators of these microenvironmental alterations, which are characterized by the presence of vascular leakiness, ECM remodeling, metabolic reprogramming of stromal cells, and immune cell recruitment and adaptation, among other changes [94]. For instance, EVs secreted by highly metastatic tumor cells educate bone marrow progenitors toward a provasculogenic and prometastatic phenotype through the transfer of Met oncoprotein, promoting their recruitment to lung PMNs and favoring metastatic colonization [54]. Importantly, tumor‐derived EVs can prepare the PMN in an organ‐specific manner due to the expression of specific integrins on their surface, thereby determining organotropic metastasis [95]. Similarly, in CRC model, the uptake of integrin beta‐like 1 (ITGBL1)‐rich EVs by resident fibroblasts in the lung and liver promote activation to CAFs, which produce proinflammatory cytokines (IL‐6, IL‐8) leading to PMN formation and metastatic growth [96]. Furthermore, novel insights regarding organotropic EV biogenesis have been described. Particularly, GTPases of the Ral family (A and B) regulate multivesicular bodies homeostasis and modulate the biogenesis and secretion of pro‐metastatic EVs by promoting MCAM/CD146 adhesion molecule loading, which favors efficient lung targeting and PMN priming, and finally sustains efficient metastasis [97]. These studies underline the potential use of EVs to predict organ‐specific metastasis and provide attractive therapeutic interference pathways to treat metastatic disease.

Although understanding distal metastatic formation is of great importance to allow the development of novel treatments that target metastasis, special attention should also be paid to the initial dissemination of cancer cells to sentinel lymph nodes, which usually are first colonized. In this sense, a recent study by García‐Silva et al. showed that melanoma‐derived sEVs enhance lymphangiogenesis and lymph node metastasis through the transfer of NGFR protein. Indeed, sEVs are uptaken by lymphatic endothelial cells activating ERK and NF‐κB pathways and ICAM‐1 expression, which reinforce tumor cell adhesion [98].

Furthermore, preclinical studies blocking EV biogenesis and secretion have been performed through the inhibition of regulators of membrane trafficking and EV formation such as Rab27a, RalA, or RalB, leading to a decrease of metastasis in *in vivo* experiments [54, 97, 99, 100]. Downregulation of RAB22A, which is also involved in the formation, trafficking, and metabolism of exosomes, diminishes exosome‐mediated breast cancer cell proliferation, invasion, and migration suggesting a similar effect as the previously cited proteins [101]. Likewise, pharmacological blockade of EV uptake leads to the reversal of PMN formation and inhibition of metastasis [102]. These results suggest that specific treatments targeting tumor EV secretion and stromal cell uptake could be promising mechanisms exploited for therapy.

The engineering of exosomes with a specific cargo has also been proposed as a possible mechanism targeting tumor cells. Chemotherapeutic agents together with interference RNAs are some of the molecules that can be loaded into EVs for cancer treatment. Indeed, preclinical studies have observed a reduction in tumor progression and increased overall survival of mice after treatment with exosomes exogenously loaded with siRNA specific to the tumor‐driver KrasG12D mutation [103]. Similarly, other authors described anti‐cancer effects in rodents treated with modified EVs through a miRNA‐dependent mechanism [104, 105] or through the delivery of paclitaxel [106, 107], suggesting that this novel therapeutic approach could become a reality in clinical practice in the near future. Indeed, the advances made in this area have encouraged the development of some clinical trials such as a phase I clinical trial (ID: NCT03608631) assessing the best dose and side effects of modified EVs containing siRNAs for treating pancreatic cancer patients with KrasG12D mutation, or a study (ID: NCT04131231) evaluating the safety and effectiveness of microparticles loaded with chemotherapeutic drugs on the treatment of malignant pleural effusion in advanced lung or breast cancer patients. The results of this last trial in lung adenocarcinoma patients showed low‐grade toxicity and suggested clinical benefits, killing tumor cells and tumor‐associated macrophages together with inducing antitumor immune responses [108], giving hope for the use of EV‐based alternative therapies for cancer patients. Still, multiple difficulties (e.g., EV isolation methodology standardization, upscale EV production, etc) need to be first discerned.

## 5 RNAs in EVs, modulators of recipient cells phenotype

The presence of functional RNA in EVs was first described by Ratajczak et al. in 2006. They observed that the treatment of hematopoietic progenitor cells with membrane‐derived vesicles from embryonic stem cells resulted in the horizontal transfer of mRNA and the translation into the corresponding proteins [109]. Since then, an increasing number of studies have analyzed the presence of different types of RNAs in EVs and their implication in recipient cells phenotype.

Extracellular vesicles can contain intact mRNA [110], mRNA fragments [111, 112] and a wide variety of noncoding RNAs including microRNAs (miRNAs) [112, 113, 114], long non‐coding RNAs (lncRNAs) [112, 115], circular RNAs (circRNAs) [116, 117], rRNAs [112], tRNAs [112, 118], vault RNA [118], SRP‐RNA [118], Y‐RNA [118], small nucleolar RNA [112, 114], small nuclear RNAs [112, 114] and piwi‐interacting RNAs [112]. Once in the recipient cells, these RNA populations can regulate gene expression processes or serve as templates for protein translation, leading to the alteration of cell function. Importantly, RNAs are not passively loaded into EVs. Indeed, specific populations of RNAs can be selectively exported becoming enriched in EVs relative to cellular RNA, which suggests the existence of RNA sorting mechanisms [119]. Furthermore, RNA profiles can differ among different subtypes of EVs, with rRNAs being more prominent in apoptotic bodies and small RNAs enriched in exosomes [120].

In cancer, EV‐associated RNAs play a crucial role in different processes contributing to cancer progressions such as the induction of angiogenesis [121, 122], acquisition of chemoresistance by sensitive tumor cells [123, 124], or the induction of EMT [125, 126]. For example, recent studies have demonstrated that tumor EVs contribute to angiogenesis by the horizontal transfer of miRNAs. The release of miR‐92a and miR‐210 enriched exosomes from leukemic and lung adenocarcinoma cells, respectively, enhance endothelial cell migration and tube formation [121, 122]. In addition, exosomal miRNA and other noncoding RNAs represent a novel mechanism of chemoresistance gained by sensitive cells. Drug‐resistant breast cancer cells may spread resistance capacity to sensitive ones by releasing exosomes carrying specific miRNAs (e.g., miR‐222) that target PTEN, activating PI3K pathway and stimulating cell proliferation and resistance, although the specific mechanism by which cells become resistant has not been elucidated [124]. Furthermore, a stress‐responsive lncRNA (lincRNA‐ROR) highly expressed in hepatocellular cancer cells and enriched in EVs derived from tumor cells may induce acquired chemoresistance within tissues through horizontal transfer [123]. Exosomes derived from CAFs and their miRNAs (miRs ‐21, ‐378e, and ‐143) can also induce stemness and EMT phenotypes in different breast cancer cell lines [125], manifesting the wide range of effects and phenotypic changes induced by EV‐derived RNAs.

Apart from lncRNAs and miRNAs, novel types of RNA have been recognized as important players in cancer development and progression. circRNAs are a special class of covalently closed noncoding RNAs generated by a process of back‐splicing [127]. They are enriched in exosomes (exo‐circRNAs) compared with the parental cells and have been identified as new cancer biomarkers, enabling the discrimination between cancer patients and healthy controls [116]. Moreover, the biological function of exo‐circRNAs may be retained and transmitted to recipient cells [116]. Increasing studies are describing new roles of exo‐circRNAs in cancer, including proliferation and invasion of tumor cells [128]. For instance, circSATB2 is highly expressed in non‐small cell lung carcinoma (NSCLC) cells and tissues and can be transferred by exosomes promoting the proliferation, migration, and invasion of NSCLC cells along with an abnormal proliferation of normal human bronchial cells [129]. In line with these data, exosomal circ‐PDE8A is associated with tumor progression and lymphatic invasion by the miR‐338/MACC1/MET pathway in pancreatic cancer [130]. Furthermore, in ovarian cancer, mice injected with exosomes containing circWHSC1 presented increased peritoneal metastasis compared with control mice by acting on peritoneal mesothelium [131]. Still, although some insights regarding the role of exo‐circRNAs have been described, their specific functions and implications in cancer remain largely unexplored.

Importantly, RNAs carried in EVs have also been proposed as drivers of oncogenic transformation [132]. Particularly, Zeng et al. [132] observed that EVs facilitated the neoplastic growth of pretransformed astrocytes, inducing proliferation, self‐renewal, and colony formation via metabolic reprogramming, and proposed the horizontal transfer of mRNAs as a possible mechanism enabling the shift in astrocyte metabolism and promoting neoplastic transformation. Furthermore, full coding‐sequence oncogene mRNAs such as *CMYC* or *CCND3* can be encapsulated in glioma‐derived EVs, which may be an additional mechanism contributing to cell transformation [132]. It is important to mention that this oncogenic transformation needs the combinatorial action of both EV‐related factors and previous oncogene‐induced pretransformed environment, suggesting that EVs have a limited action. In line with these data, exosome‐like EVs containing genomic DNA, H‐ras oncoprotein, and transcript can be internalized by fibroblasts inducing phenotypic transformation; however, these effects are transient, failing to complete oncogenic transformation [133]. Together, these results suggest that EVs could act in combination with other modifications to promote cell transformation. Apart from that, EVs containing miR‐424 from advanced prostate cancer patients induced the acquisition of stem‐like features in low tumorigenic cells, contributing to disease progression and suggesting that miRNAs can also support the acquisition of cell aggressiveness by indolent tumor cells, propagating oncogenic stimuli [134]. Importantly, EVs isolated from MDA‐MB‐231 breast cancer cells and serum from breast cancer patients are able to stimulate nontumorigenic MCF10A mammary epithelial cells to form tumors altering the transcriptome of recipient cells in a Dicer‐dependent manner [135]. Moreover, the horizontal transfer of circRNA_100284 via EVs leads to an accelerated cell cycle and increased proliferation of L‐02 normal cells [136]. All these data suggest the possible role of different types of RNA carried in EVs as promoters of the malignant transformation of neighboring and distant cells.

## 6 Role of EVs in chromosomal rearrangements and genomic instability

Genomic instability is a characteristic of most cancers. Deficiency in DNA repair mechanisms, DNA damage, or mitotic checkpoints together with oncogene‐induced DNA replication stress, contribute to mutational processes ranging from base pair substitutions to chromosomal rearrangements that occur in cancer genomes and can drive tumor initiation and/or progression [137].

Extracellular vesicles have been proposed to promote genomic alterations in recipient cells. Besides, recent evidence showed that EVs from tumor cells induce genomic instability in cells from the surrounding microenvironment. Particularly, Dutta et al. described that exosomes released by breast cancer cell lines (MDA‐MB‐231, T47DA18, and MCF7) are taken up by human primary mammary epithelial cells (HMECs) inducing the generation of DNA damage repair responses, p53 phosphorylation, and autophagy, probably due to induction of reactive oxygen species. In turn, HMECs release breast cancer cell growth promoting factors, suggesting that breast cancer cell‐derived EVs promote the formation of a tumor‐supportive microenvironment [138]. Furthermore, tumor‐derived‐EVs can also induce genome instability together with altered morphology and cell structures, loss of cell–cell contact inhibition, and increased invasiveness in nonmalignant human SV‐HUC urothelial cells through a mechanism directly related to endoplasmic reticulum stress‐induced unfolded protein response [139]. In line with these data, cancer cell‐derived EVs contribute to pathological vascular responses in endothelial cells including angiogenesis‐like migratory responses and formation of micronuclei, as a result of the intercellular transmission of genomic instability, suggesting its contribution in tumor‐associated aberrations of the vascular growth [140].

Apart from microenvironmental cells, alterations can also be promoted in naïve tumor cells. Interestingly, heat‐shocked MCF7 breast cancer cell‐derived EVs can induce DNA damage and apoptosis in naive MCF7 cells together with cell invasion [141], suggesting that genomic alterations and aggressiveness can be transferred among tumor cells. Furthermore, the presence of retrotransposon RNA transcripts (HERV, LINE‐1, Alu elements) in tumor‐derived EVs represent another mechanism by which genomic instability could be altered in recipient cells; however, it is currently unknown whether these transposable elements can insert into the target genome [142].

Another interesting fact is that EVs can carry genomic DNA spanning all chromosomes, including mutations in oncogenes such as *KRAS*, *TP53* [143], or *HRAS* [144], which can be taken up by cells not carrying these mutations [144]. Moreover, an increasing number of studies are reporting the use of EVs as potential biomarkers of chromosomal rearrangements in different types of cancer [145], which suggests that information regarding genomic abnormalities occurring in tumor cells can be detected and transferred to other cell types through horizontal transfer. In line with these data, the EV‐dependent transfer of DNA has been described to induce phenotypic changes in recipient cells. For instance, DNA from *HRAS*V12 and *CMYC* transfected cells can be transmitted to p53‐deficient cells inducing loss of contact inhibition *in vitro* and tumor formation *in vivo* [146]. Together, these studies bring to light the possibility that EVs could transfer mutations that can alter recipient cells and may induce oncogenic transformation.

Indeed, in the late 90s, García‐Olmo et al. [147] proposed that metastasis might develop through the transfection of target cells in distant organs with primary tumor‐derived oncogenes present in the circulating plasma. This hypothesis was later supported by Abdouh and collaborators, who proposed that metastatic disease could be due to the transformation of cells by circulating cancer factors [148] and performed different studies suggesting the role of EVs in this transformation [149, 150, 151]. Still, this hypothesis is controversial, which contradicts several principles of the cancer progression paradigm (extended review [152]).

Thus, recent advances showed that tumor‐derived EVs can induce genetic instability in recipient cells promoting a niche susceptible for tumor growth and progression. However, important questions remain unanswered such as, can EVs modify the phenotype of other tumor cells inducing specific genomic alterations (i.e., DNA damage and chromosomal rearrangements)? Are these mechanisms involved in tumor cell heterogeneity and malignancy? Or more importantly, can tumor‐derived EVs transform cells within the tumor microenvironment leading to oncogenic transformation? Additional studies must be performed to understand the role of EVs in the intercellular propagation of genomic instability triggered by tumor cells and the implication of this transformation in tumor development and progression.

## 7 Final remarks

The process of carcinogenesis has been extensively described by countless authors worldwide. In this regard, the Hallmarks of Cancer were proposed by Hanahan and Weinberg [153, 154] as a set of functional capabilities acquired by human cells that are transitioning into a malignant phenotype and are essential to finally form a tumor. The objective of these studies was to describe shared features among all cancer cell types that allow the analysis of complex tumor phenotypes with regard to intrinsic cellular parameters [153, 154]. The six initial hallmarks described [153] were later expanded to eight [154] and comprise acquired capabilities for sustaining proliferative signaling, evading growth suppressors, resisting cell death, enabling replicative immortality, inducing/accessing vasculature, activating invasion and metastasis, reprogramming cellular metabolism, and avoiding immune destruction. Furthermore, genome instability and tumor‐promoting inflammation were added as ‘enabling characteristics’ by which tumor cells can acquire these hallmark traits [154]. More recently, additional prospective emerging hallmarks and enabling characteristics have been described, including unlocking phenotypic plasticity, nonmutational epigenetic reprogramming, polymorphic microbiomes, and senescent cells [2]. Surprisingly, the role of EVs is left behind.

The knowledge regarding the role of EVs in cancer has expanded in the last years, although there is still a long road ahead to truly understand all the mechanisms and functions of EVs in primary tumor and metastasis. The study of the potential role of distinct subpopulations of EVs affecting differently tumor progression and PMN formation, the effect of tumor heterogeneity in EV secretion, or the result of continuous conditioning of tumor microenvironment and premetastatic and metastatic niches, are concepts that need to be clarified in order to gain a better understanding of the whole cancer process. Even so, the existing knowledge has already given multiple clues that could be used for cancer therapy. Indeed, inhibition of EV secretion or uptake in preclinical models has shown promising results. However, side effects could also occur due to the alteration of EV trafficking between healthy cells, being crucial to investigate possible mechanisms that specifically block vesicles from tumor cells. Engineering EVs with a specific cargo has also become a reality, although similar challenges such as specific targeting of recipient cells, standardization of purification methods, or selection of a route of administration must be overcome.

Even though numerous studies have focused on the capacity of EVs to alter the tumor microenvironment or induce changes in distal locations (e.g., PMN formation), most of the research has mostly addressed the reprogramming of stromal cells toward a tumor‐supportive phenotype. Nevertheless, there is growing data supporting that tumor‐derived EVs not only modify microenvironmental cells to better accommodate tumor cells and facilitate tumor progression but may also induce the oncogenic transformation of indolent cell types outside the primary tumor, shifting our understanding of cancer from a merely evolutionary process to an infective disease.

The possible consequences of this transformation go beyond the established knowledge of tumor development and progression and open multiple interesting questions. Can EVs facilitate tumor recurrence by the transformation or the induction of oncogenic predisposition of nearby stromal cells? Can EVs from a specific tumor type induce the malignant transformation of cells toward other molecular phenotypes or other tumor types? Can this process explain in part tumor heterogeneity? Indeed, a recent study suggests that tumor‐derived EVs can induce fibroblast heterogeneity in CRC models determined by the EMT status of the tumor cells through a miR‐200/ZEB1/TGF‐β dependent mechanism [155], opening a window to the possibility that other cell types, including tumor cells, could be also affected by similar mechanisms.

It is also plausible to speculate that the continuous conditioning of distant niches by EVs secreted from the primary tumor might lead to the formation of ‘EV‐inducible metastasis’ (Fig. 1B). This hypothesis challenges the process of metastatic dissemination—currently understood as a series of discrete steps by which tumor cells leave the primary tumor and colonize distal organs—as the only possible mechanism to induce the formation of metastasis. Furthermore, it brings to light multiple interrogations regarding the characteristics of these novel ‘EV‐inducible metastatic cells’.

In summary, growing evidence suggests that EVs may be determinant factors promoting oncogenesis; however, specific studies should be conducted to clarify this hypothesis as it holds important implications for understanding cancer as a whole.

## Conflict of interest

AR reports personal fees from Takeda and AstraZeneca, outside the submitted work. MP reports grants, personal fees, and travel expenses from BristolMyers Squibb, Roche, and AstraZeneca; and personal fees from Merck Sharpe & Dohme and Takeda, outside the submitted work. LRL, ES‐H, and RS‐B declare no conflicts of interest.

## Author contributions

LRL and AR drafted and critically revised the manuscript. ES‐H, RS‐B, and MP reviewed the draft. All authors read and approved the submitted version.
